# Die Subanalyse von Rheuma-VOR zeigt den erheblichen Bedarf der rheumatologischen Versorgung auf

**DOI:** 10.1007/s00393-024-01490-2

**Published:** 2024-03-08

**Authors:** Stefanie Hirsch, K. Hoeper, D. Meyer-Olson, A. Schwarting, K. Gente, M. Dreher, J. Hoeper, T. Witte, T. Thiele

**Affiliations:** 1 Klinik für Rheumatologie und Immunologie, Medizinische Hochschule Hannover, Carl-Neuberg-Straße 1, 30625 Hannover, Deutschland; 2 Regionales Kooperatives Rheumazentrum Niedersachsen e. V., Hannover, Deutschland; 3 m&i Fachklinik Bad Pyrmont und MVZ Weserbergland, Bad Pyrmont, Deutschland; 4 Schwerpunkt Rheumatologie und klinische Immunologie, Universitätsmedizin der Johannes Gutenberg Universität Mainz, Mainz, Deutschland; 5 Innere Medizin V– Sektion Rheumatologie, Universitätsklinikum Heidelberg, Heidelberg, Deutschland; 6 Center for Health Economics Research Hannover (CHERH), Leibniz University Hannover, Hannover, Deutschland

**Keywords:** Entzündlich-rheumatische Erkrankungen, Versorgungsdefizit, Rheumatoide Arthritis, Kollagenosen, Polymyalgia rheumatica, Inflammatory rheumatic disease, Medical care deficit, Rheumatoid arthritis, Collagenosis, Polymalgia rheumatica

## Abstract

**Hintergrund:**

Eine frühe Diagnose und Behandlung entzündlich-rheumatischer Erkrankungen kann Folgeschäden wie dauerhafte Einschränkungen der Mobilität und Gelenk- oder Organschäden verhindern. Gleichzeitig besteht ein größer werdendes Versorgungsdefizit aufgrund fehlender rheumatologischer Kapazitäten. Betroffen sind besonders die ländlichen Regionen.

**Ziel der Arbeit:**

Die vorliegenden nicht bestätigten Diagnosen der Studie Rheuma-VOR wurden hinsichtlich des Vorliegens einer anderen definitiven entzündlich-rheumatischen Erkrankung analysiert.

**Material und Methoden:**

Die eingegangenen Fragebögen der an der Rheuma-VOR-Studie teilnehmenden Rheumatolog:innen wurden nach Vermerken anderer entzündlich-rheumatischer Erkrankungen als der geforderten Diagnose einer rheumatoiden Arthritis, Psoriasis-Arthritis oder Spondyloarthritis gescreent.

**Ergebnisse:**

Von 910 „nicht bestätigten“ Diagnosen waren bei 245 Patient:innen andere gestellte Diagnosen auszuwerten. Insgesamt 29,8 % der Diagnosen entsprechen degenerativen Gelenkveränderungen oder chronischen Schmerzsyndromen, bei 26,1 % lagen verschiedene Formen entzündlicher Arthritiden vor. Der Großteil der Diagnosen (40,5 %) entfiel auf Kollagenosen und Vaskulitiden, wobei die Polymyalgia rheumatica mit 20 % am häufigsten diagnostiziert wurde (49 Patient:innen).

**Diskussion:**

Die vorliegenden Daten zeigen, dass bei einem Großteil der Patient:innen die rheumatologische Vorstellung indiziert war. Aufgrund der ambulanten Versorgungsdefizite ist eine vorherige Selektion des Patientenguts essenziell, um die eingeschränkten Kapazitäten bestmöglich zu nutzen.

## Abstract (en)

**Background:**

Early diagnosis and treatment of inflammatory rheumatic diseases can prevent consequential damage such as permanently limited mobility and joint or organ damage. Simultaneously, there is an increasing deficit in medical care owing to the lack of rheumatological capacity. Rural regions are particularly affected.

**Objectives:**

The available unconfirmed diagnoses of the study Rheuma-VOR were analysed regarding another definitive inflammatory rheumatic disease.

**Materials and methods:**

The returned questionnaires of the rheumatologists participating in Rheuma-VOR were screened for definitive inflammatory rheumatic diseases other than the required diagnosis of rheumatoid arthritis, psoriatic arthritis or spondyloarthritis.

**Results:**

Of 910 unconfirmed diagnoses, in 245 patients another definitive diagnosis could be confirmed. A total of 29.8% of the diagnoses corresponded to degenerative joint changes or chronic pain syndrome, whereas 26.1% involved different forms of inflammatory arthritis. The majority of diagnoses (40.5%) were collagenosis or vasculitis,

**Discussion:**

The available data show that a rheumatological presentation was indicated for the majority of patients. Owing to the increasing deficits in medical care a prior selection of the patients is crucial to make optimal use of restricted rheumatological capacities.

Die Frühdiagnose und rasche Behandlung entzündlich-rheumatischer Erkrankungen kann in vielen Fällen langfristige Gelenkschäden und Mobilitätseinschränkungen reduzieren oder sogar verhindern [1]. Demgegenüber steht jedoch ein erhebliches rheumatologisches Versorgungsdefizit. Vor allem in den ländlichen Regionen fehlen internistische Rheumatolog:innen zur adäquaten Versorgung.

## Hintergrund

Es existieren bereits diverse Modelle von Früh- und Screeningsprechstunden [2], um die Zeit vom Auftreten erster Symptome bis zur Diagnose und Therapieeinleitung zu verkürzen. Es liegt nahe, dass regionale Faktoren einen Einfluss auf das Patientenkollektiv und somit auf die Struktur der Früharthritissprechstunde haben könnten.

In den letzten Jahren wurden enorme Fortschritte in der Behandlung der rheumatologischen Erkrankungen erzielt. So kann bei frühzeitiger Diagnosestellung der rheumatoiden Arthritis der Verlauf günstig beeinflusst werden. Eine lang anhaltende Remission (kein Nachweis einer Krankheitsaktivität) ohne Auftreten von knöchernen Erosionen oder Endorganschäden ist potenziell möglich. Somit kommt der rechtzeitigen Diagnosestellung eine große Bedeutung zu. Bei einem verzögerten Behandlungsbeginn aufgrund des massiven Versorgungsdefizites können aber bereits irreversible Schäden im Bereich der Gelenke und der Organe aufgetreten sein [3].

Die aus ADAPTHERA weiterentwickelte und auf mehrere Bundesländer erweiterte prospektive multizentrische Netzwerkstudie „Rheuma-VOR“ verfolgte das Ziel, drei der am häufigsten vorkommenden rheumatologischen Erkrankungen frühzeitig zu erkennen und die Versorgungsqualität der Behandlung zu verbessern [4]. Die Ergebnisse der abgeschlossenen Studie sind kürzlich veröffentlicht worden [5]. Aktuelle Schätzungen gehen davon aus, dass allein in Deutschland bis zu 1,2 Mio. Menschen an einer rheumatoiden Arthritis (RA), einer Psoriasis-Arthritis (PsA) oder einer axialen Spondyloarthritis (axSpA) leiden [6]. Die Latenz zwischen dem Auftreten erster Symptome bis zur Diagnosestellung beträgt bei der rheumatoiden Arthritis ein Jahr, bei der Psoriasis-Arthritis drei Jahre und bei der axialen Spondyloarthritis sogar fünf Jahre [7]. Das sogenannte „window of opportunity“, in dem eine zielgerichtete Therapie (Treat-to-Target-Prinzip) den Verlauf und das Outcome der Erkrankung maßgeblich verbessern kann, liegt aber wahrscheinlich in den ersten Monaten nach Erkrankungsbeginn [8]. Die Verzögerung der Diagnosestellung kann zu enormen sozioökonomischen Folgekosten, u. a. durch frühzeitige Erwerbsminderung oder Berentung, führen [9].

Die frühzeitige Detektion teils akut verlaufender systemischer Erkrankungen aus dem Bereich der Kollagenosen und Vaskulitiden war nicht primäres Ziel der Studie Rheuma-VOR. Jedoch wurden die Screeningbögen für Rheuma-VOR häufig unabhängig der angegebenen Fragen individuell vom zuweisenden Arzt:in ergänzt. Neben erhöhten ANA-Titern (> 1:2560) wurden auch Symptome wie Raynaud-Syndrom, Gesichtsrötungen, Gewichtsverlust, Fieber und vaskulitische Effloreszenzen vermerkt. Da diese Symptome und Laborparameter Hinweis für eine zugrunde liegende entzündliche Systemerkrankung sein können, wurden auch diese Patient:innen zur Sprechstunde einbestellt, um einen möglichen gefährlichen Verlauf rechtzeitig zu erkennen bzw. abzuwenden.

## Projekt

**Abb. 1:**
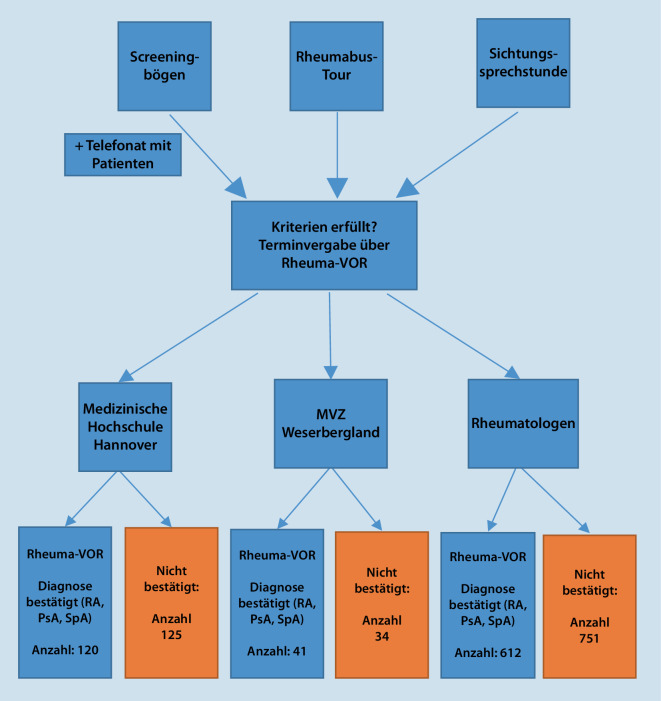
Ablaufplan des Projekts Rheuma-VOR in Niedersachsen

Kernansatz des Netzwerks Rheuma-VOR ist das Konzept der koordinierten Kooperation zwischen Primärversorger:innen, Rheumatolog:innen, den Kliniken und den jeweiligen Rheumazentren. Primärversorger:innen konnten einen ein- bis zweiseitigen Screeningbogen mit einer der drei Verdachtsdiagnosen (RA, PsA, axiale SpA) ausfüllen. Diese Bögen wurden in der bundeslandspezifischen Koordinationsstelle gesichtet. In Niedersachen erfolgte zusätzlich zum Faxeingang des Screeningbogens ein Telefonat mit den Patient:innen, um weitere Leitsymptome zu erfassen. Bei hochgradigem Verdacht auf eine zugrunde liegende rheumatische Erkrankung erfolgte die Überweisung der Patient:innen zu an Rheuma-VOR teilnehmende niedergelassene Rheumatolog:innen, das MVZ Weserbergland in Bad Pyrmont oder an die rheumatologische Ambulanz der Medizinischen Hochschule Hannover (Abb. 1).

Im Rahmen des Rheuma-VOR-Projekts erfolgte während der Projektlaufzeit in Niedersachsen zweimal (2018 und 2019) eine Rheumabus-Tour als „Open-Access-Screening-Veranstaltung“. Die für 2020 geplante Tour musste pandemiebedingt abgesagt werden. Angesteuert wurden primär Ortschaften im Norden Niedersachsens. Ziel war die Früherkennung der drei rheumatologischen Erkrankungen RA, PsA und axiale SpA. Insgesamt wurden mehr als 400 Patient:innen bei den Touren gescreent, für 139 Patient:innen wurde bei Verdacht auf eine aktive rheumatische Erkrankung ein kurzfristiger Termin zur weiteren rheumatologischen Abklärung vermittelt.

Als weiteres Mittel zur niederschwelligen Patientenrekrutierung erfolgte zur Vermeidung langer Anfahrtswege für die Patient:innen eine monatliche Sichtungssprechstunde sowohl im MVZ Weserbergland als auch in den Räumlichkeiten der Rheumatologie des Nord-West Krankenhauses Sanderbusch. Terminanfragen aus der Region Bad Pyrmont wurden im MVZ im Rahmen einer Screeningsprechstunde selektioniert. Patient:innen mit Terminanfragen im Krankenhaus Sanderbusch bzw. Terminanfragen an die Rheuma-VOR-Koordinationsstelle aus den nördlichen Regionen Niedersachsens wurden in Sanderbusch in einem 15-minütigen Termin von einer rheumatologischen Ärztin mittels Anamnese und fokussierter klinischer Untersuchung abgeklärt. Im Rahmen beider Screeningsprechstunden erfolgte bei Verdacht auf das Vorliegen einer der drei Erkrankungen die Weiterleitung zu einem fachärztlichen Termin in einer der rheumatologischen Kooperationseinrichtungen.

Sowohl bei den eingegangenen Screeningbögen der Primärversorger:innen, der Rheumabus-Tour als auch bei den Sichtungssprechstunden in Sanderbusch und Bad Pyrmont ergaben sich auch Verdachtsdiagnosen auf eine andere als der drei für das Projekt Rheuma-VOR geforderten rheumatischen Erkrankungen RA, PsA oder axSpA.

Bei Kollagenosen, Vaskulitiden und der Polymyalgia rheumatica stellt eine zeitnahe Diagnose und Einleitung einer Behandlung ebenfalls einen wichtigen Faktor für den weiteren Verlauf der Erkrankung dar. Aus diesem Grund erfolgten auch bei diesen Angaben Terminvergaben, um schwere Verläufe einer Erkrankung abzuwenden. Die gestellten Diagnosen waren nicht primärer Endpunkt der Datenerhebung des Projekts Rheuma-VOR. Konnte keine der drei geforderten Diagnosen (RA, PsA, SpA) gestellt werden, zählten die Patient:innen im Rahmen des Projektes als „nicht bestätigte“ Fälle. In Niedersachsen konnte ein Teil der „nicht bestätigten“ Fälle aufgearbeitet werden.

## Ergebnisse

Während des laufenden Projektes gingen in Niedersachsen 2849 Screeningfaxe ein. Bei 1915 der eingegangenen Anfragen (67,2 %) bestand nach der Auswertung der Screeningfragen des Bogens der Verdacht auf das Vorliegen einer entzündlich-rheumatischen Erkrankung, sodass ein Termin bei einer Rheumatolog:in vereinbart wurde. Die Wartezeit vom Eingang des Screeningbogens bis zum rheumatologischen Facharzttermin lag in Niedersachsen bei durchschnittlich 34 Tagen. Insgesamt sind 232 Patient:innen (12 %) nicht zum vereinbarten Termin erschienen.

Bei den 1915 vereinbarten Terminen konnte bei 773 Patient:innen die Diagnose einer RA (*n* = 496), PsA (*n* = 158) oder axSpA (*n* = 119) gestellt werden.

Bei den 910 „nicht bestätigten“ Diagnosen liegen uns Daten bzw. andere Diagnosen von 245 Patient:innen vor (Tab. 1).

**Tab. 1:** Vom Rheumatologen gestellte Diagnosen der „nicht bestätigten“ Rheuma-VOR-Vorstellungen

	Medizinische Hochschule Hannover	MVZ Weserbergland	Schwerpunkt Rheumatologen	Insgesamt
*Entzündlich-rheumatische Systemerkrankungen*				
Sarkoidose und/oder Löfgren-Syndrom	2	0	2	4
Kollagenose/undiff. Kollagenose	8	6	8	22
Sjögren-Syndrom	10	0	2	12
Systemischer Lupus erythematodes	0	6	0	6
Overlap	0	0	1	1
Limitierte systemische Sklerodermie	1	0	0	1
Diffuse systemische Sklerodermie mit Lungenbeteiligung	0	0	1	1
Vaskulitis, nicht näher bezeichnet	1	0	1	2
Granulomatose mit Polyangiitis (GPA)	0	1	0	1
Großgefäßvaskulitis	0	1	0	1
Polymyositis	1	0	0	1
Dermatomyositis	0	0	1	1
Antisynthetase-Syndrom	1	0	0	1
Polymyalgia rheumatica	12	10	27	49
*Formen entzündlich-rheumatischer Gelenkerkrankungen*				
Seronegative rheumatoide Arthritis	0	8	2	10
Arthritis psoriatica, sonstige Form	0	1	2	3
Arthritis, nicht näher bezeichnet	3	1	9	13
Monarthritis	1	0	1	2
Oligoarthritis	1	0	0	1
Undifferenzierte Spondyloarthritis	4	2	2	8
Sakroiliitis, nicht näher bezeichnet	1	0	4	5
Enteropathische Arthritis	3	0	0	3
Reaktive Arthritis	4	0	6	10
Virus assoziierte Arthritis	0	0	2	2
V. a. Lyme-Arthritis	1	0	1	2
Palindromer Rheumatismus	0	0	1	1
Arthritis urica	0	0	4	4
**Diagnosen: Degenerative Befunde/Schmerzen:**	22	0	51	73
Andere Erkrankungen	3	0	2	5
**Gesamt**	**79**	**36**	**130**	**245**

Bei 73 der 245 Patient:innen (29,8 %) konnte eine Diagnose gestellt werden, die entweder einer degenerativen Gelenkerkrankung entsprach, oder die Kriterien für ein chronisches generalisiertes Schmerzsyndrom erfüllte. Bei 64 Patient:innen (26,1 %) wurden verschiedene Formen von Arthritiden und Spondyloarthritiden diagnostiziert, die nicht der geforderten ICD-10-Diagnose für Rheuma-VOR entsprachen.

**Abb. 2:**
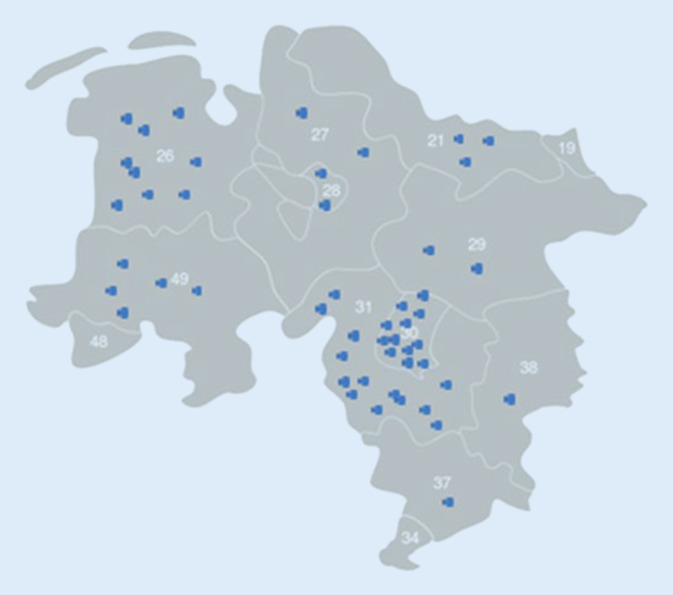
Schematische Darstellung Bundesland Niedersachsen mit Postleizahlen. Jeder *blaue Punkt* auf der Karte entspricht der Diagnose Polymyalgia rheumatica (Postleitzahl des Patienten)

Am häufigsten konnten Diagnosen aus dem Formenkreis der Kollagenosen und Vaskulitiden gestellt werden (40,5 %). Bei 22 Patient:innen wurde die Diagnose einer undifferenzierten Kollagenose gestellt, in 12 Fällen konnte die Diagnose eines Sjögren-Syndroms gestellt werden, ein systemischer Lupus erythematodes wurde sechsmal diagnostiziert. Jeweils einmal erfolgte die Diagnose Polymyositis, Dermatomyositis, Antisynthetase-Syndrom, Großgefäßvaskulitis, systemische Sklerose mit Lungenbeteiligung und Granulomatose mit Polyangiitis. Den größten Anteil der rheumatologischen Diagnosen stellte die Polymyalgia rheumatica dar. Diese wurde bei 49 Patient:innen (20 %) diagnostiziert (Abb. 2).

## Diskussion

Im Rahmen des Projekts Rheuma-VOR wurden in Niedersachsen 1915 Termine vermittelt. In 773 Fällen (40,4 %) der Vorstellungen konnte die Diagnose einer RA, PsA oder SpA gestellt werden. Das Hauptaugenmerk der vorliegenden Analyse galt den 910 „nicht bestätigten“ Diagnosen.

In knapp 27 % der Fälle (245 Patient:innen) liegt uns die gestellte Diagnose der „nicht bestätigten“ Diagnose vor. Bei den 245 Patient:innen entsprechen 163 Diagnosen (66,5 %) eindeutigen rheumatologischen Erkrankungen (verschiedene Formen der Arthritiden, Spondyloarthritiden, Kollagenosen und Vaskulitiden). Es ist anzunehmen, dass auch bei den 665 nicht vorliegenden Diagnosen der 910 „nicht bestätigten“ Diagnosen eine ähnliche Verteilung bestehen könnte.

Die vorliegenden Daten zeigen, dass bei einem Großteil der Patient:innen die Vorstellung bei einer Rheumatolog:in indiziert war. Trotz der schnellen Terminvergabe (im Durchschnitt 34 Tage) erschienen 232 Patient:innen (12 %) nicht zu dem vereinbarten Termin. Häufigster Absagegrund war der lange Anfahrtsweg zu Rheumatolog:innen bei älteren immobilen Patient:innen.

Die große Anzahl der Faxeingänge mit freihändigen Notizen der Primärversorger:innen und die große Anzahl an Diagnosen aus dem rheumatischen Formenkreis zeigen ein großes strukturelles Versorgungsproblem auf. Zwar haben sich aufgrund der knappen rheumatologischen Versorgungskapazitäten regionale Versorgungsnetzwerke mit schneller, kollegialer, fachärztlicher Zuweisung bereits gebildet, diese scheinen aber eher in Gebieten mit praktizierenden Rheumatolog:innen zu funktionieren. In Landkreisen ohne Rheumatolog:innen ist die schnelle Zuweisung von Kolleg:in zu Kolleg:in deutlich weniger praktikabel. Hier gilt es langfristig eine Lösung zu schaffen. Die Prävalenz der Polymyalgia rheumatica steigt mit zunehmendem Alter an. Die Diagnose ist für Rheumatolog:innen anhand der typischen Symptome wie Schwere im Schulter- und Beckengürtel und deutlich erhöhten systemischen Inflammationsparametern und gelegentlich auch Allgemeinsymptomen wie Gewichtsverlust, Nachtschweiß und Fieber rasch zu stellen. Eine mögliche begleitende Großgefäßvaskulitis, die in 5–30 % der Fälle auftritt [10, 11], wird bei Verdacht durch den Rheumatolog:in mittels bildgebender Diagnostik weiter abgeklärt. Auch eine „late-onset“ rheumatoide Arthritis muss differentialdiagnostisch abgeklärt werden. Inwieweit dies bei den 49 Patienten mit der Diagnose der Polymyalgia rheumatica der Fall war lässt sich anhand der vorliegenden Daten nicht nachvollziehen und stellt somit eine Limitation der Arbeit dar. Aufgrund der eingeschränkten ambulanten rheumatologischen Versorgungskapazitäten in Niedersachsen, mit Ballungen im Umkreis einiger Städte und komplett fehlenden Rheumatolog:innen in einzelnen Landkreisen, wird häufig proklamiert, dass die Polymyalgia rheumatica auch durch die Primärversorger:innen behandelt werden kann.

Dass im Rahmen des Projekts Rheuma-VOR bei 49 Patient:innen die Diagnose einer Polymyalgia rheumatica gestellt wurde, zeigt jedoch, dass es keine Selbstverständlichkeit ist, dass diese Erkrankung durch Primärversorger:innen erkannt und behandelt wird. Die für Rheumatolog:innen häufige Diagnose der Polymyalgia rheumatica ist für manche niedergelassene Primärversorger:innen eine Erkrankung, die sie nur sehr selten sehen. Entsprechend groß kann die Unsicherheit sein möglicherweise eine andere rheumatologische Erkrankung zu übersehen und so den Verlauf durch einen nicht rechtzeitigen Behandlungsbeginn zu verschlechtern. Die Sorge ist nicht gänzlich unbegründet, da die Polymyalgia rheumatica in 5–30 % der Fälle mit einer Großgefäßvaskulitis assoziiert sein kann.

Diese Verläufe gilt es rechtzeitig zu detektieren. Auch die differentialdiagnostische „late-onset“ rheumatoide Arthritis muss unterschieden werden. Die Auswertung zeigt, dass der Bedarf an rheumatologischen Vorstellungen hoch ist. Und bei einem Großteil der Zuweisungen ist die Vorstellung zur Abklärung einer möglichen begleitenden Großgefäßvaskulitis oder differentialdiagnostischen „late-onset“ rheumatoiden Arthritis berechtigt.

Bei den knappen Ressourcen stellen das häufig diagnostizierte Schmerzsyndrom und die Arthrose Erkrankungen dar, die im Setting der Primärversorger:innen eventuell mithilfe von Tools und Fragebögen besser detektiert werden können, um diese Patient:innen nicht primär in den Engpass der rheumatologischen Vorstellung mit einzubinden, sondern direkt eine schmerzmedizinische Vorstellung zu bahnen.

Es wurde 22 Mal die Diagnose einer undifferenzenzierten Kollagenose gestellt. In der Hochschulambulanz wurden die Diagnosen bei erhöhten ANA-Titern (> 1:640) und klinischen Symptomen (z. B. Arthritis, Sicca-Symptomatik, Raynaud, vaskulitische Effloreszenzen, auffällige Kapillarmikroskopie), welche vereinbar mit dem Vorliegen einer Kollagenose sind, bei bis dahin noch negativem ENA gestellt. Jedoch handelt es sich bei der undifferenzierten Kollagenose nicht um validierte Klassifikationskriterien, sodass die Diagnosestellung in anderen Einrichtungen nicht derselben entsprechen muss. Dies stellt eine weitere Limitation der Arbeit da, da nicht gesichert ist, dass in einer anderen Praxis ebenfalls die Diagnose einer undifferenzierten Kollagenose gestellt worden wäre.

Solange die Kapazitäten mit schnellen rheumatologischen Vorstellungen weiterhin knapp sind, stellt eine gute Selektion der Patient:innen durch die Primärversorger:innen die wichtige Weiche für die Weiterversorgung dar.

Die ländlichen Strukturen und die weiten Anfahrtswege erschweren insbesondere bei den älteren, immobilen Patient:innen eine rechtzeitige Diagnosestellung und Behandlungsbeginn. Die durchgeführten Rheumabus-Touren in verschiedenen Städten Niedersachsens fanden großen Anklang. Für immobile Patient:innen sinkt die Hemmschwelle, sich im Heimatort ohne vorherige Terminabsprache zur Screeninguntersuchung beim geparkten Rheumabus vorzustellen. Für die regelmäßige Versorgung vom immobilen Patient:innen mit notwendiger Immunsuppression bei rheumatischer Erkrankung sind in der Zukunft möglicherweise auch Modelle der rollenden rheumatologischen Arztpraxis zu diskutieren bei mangelnder Rheumatologendichte in der Region.

Die Telemedizin kann perspektivisch zu einer Verbesserung der Versorgung beitragen. Ein Erstkontakt von Patient:in und Rheumatolog:in kann jedoch aufgrund der notwendigen klinischen Untersuchung mit Erhebung des Gelenkstatus (am Beispiel der entzündlich-rheumatischen Gelenkerkrankungen) schwerlich durch einen Videokontakt ersetzt werden. Folgetermine oder Routinekontrolltermine wären mithilfe der Telemedizin jedoch möglich. Zusammenfassend zeigen die Daten unserer Subanalyse, dass durch Rheuma-VOR, auch die (Früh‑)Diagnose anderer entzündlicher Erkrankungen verbessert und die fachärztliche Zuweisung beschleunigt werden könnte, auch wenn dieses Programm nicht primär auf diese Erkrankungen abgezielt hat.

## Fazit für die Praxis

- Das rheumatologische Versorgungsdefizit in ländlichen Regionen wird sich aufgrund des demografischen Wandels in den kommenden Jahren verstärken
- Screeningbögen für Frühsprechstunden sollten modifiziert und optimiert werden mit zusätzlicher Erfassung von Kollagenose und Vaskulitis typischen Symptomen, B‑Symptomatik und Bestimmung der Entzündungswerte
- Optimierte Ausschöpfung der eingeschränkten Ressource der rheumatologischen Vorstellung bei besserer Vorselektion des Patientenguts

## References

[1] Zink A et al (2017) Memorandum der Deutschen Gesellschaft für Rheumatologie zur Versorgungsqualität in der Rheumatologie – Update 2016. Z Rheumatol 76(3):195–207. 10.1007/s00393-017-0297-1 28364218 10.1007/s00393-017-0297-1

[2] Benesova K et al (2019) Früh- und Screeningsprechstunden: Ein notwendiger Weg zur besseren Frühversorgung in der internistischen Rheumatologie? Z Rheumatol 78(8):722–742. 10.1007/s00393-019-0683-y 31468170 10.1007/s00393-019-0683-y

[3] Smolen JS et al (2020) EULAR recommendations for the management of rheumatoid arthritis with synthetic and biological disease-modifying antirheumatic drugs: 2019 update. Ann Rheum Dis 79(6):685. 10.1136/annrheumdis-2019-216655 31969328 10.1136/annrheumdis-2019-216655

[4] Lauter A et al (2019) ADAPTHERA – Landesweit transsektorales Versorgungsnetzwerk für Patienten mit früher rheumatoider Arthritis zeigt anhaltende Remissionen in der Regelversorgung. Z Rheumatol 78(7):660–669. 10.1007/s00393-019-0653-4 31165251 10.1007/s00393-019-0653-4

[5] Dreher M et al (2023) Rheuma-VOR study: optimising healthcare of rheumatic diseases by multiprofessional coordinating centres. Ann Rheum Dis. 10.1136/ard-2023-224205 10.1136/ard-2023-224205 PMC10850684 37890976

[6] Schwarting A, Dreher M, Assmann G, Witte T, Hoeper K, Schmidt RE (2019) Erfahrungen und Ergebnisse aus Rheuma – VOR. Z Rheumatol 78(8):743–752. 10.1007/s00393-019-00694-1 31468168 10.1007/s00393-019-00694-1

[7] Albrecht K et al (2017) Versorgung der rheumatoiden Arthritis 2014. Z Rheumatol 76(1):50–57. 10.1007/s00393-016-0156-5 27379740 10.1007/s00393-016-0156-5

[8] Kerschbaumer A et al (2020) Efficacy of pharmacological treatment in rheumatoid arthritis: a systematic literature research informing the 2019 update of the EULAR recommendations for management of rheumatoid arthritis. Ann Rheum Dis 79(6):744–759. 10.1136/annrheumdis-2019-216656 32033937 10.1136/annrheumdis-2019-216656 PMC7286044

[9] Mau W, Thiele K, Lamprecht J (2014) Trends der Erwerbstätigkeit von Rheumakranken. Z Rheumatol 73(1):11–19. 10.1007/s00393-013-1205-y 24402233 10.1007/s00393-013-1205-y

[10] Gran JT, Myklebust G, Wilsgaard T, Jacobsen BK (2001) Survival in polymyalgia rheumatica and temporal arteritis: a study of 398 cases and matched population controls. Baillieres Clin Rheumatol 40(11):1238–1242. 10.1093/rheumatology/40.11.1238 10.1093/rheumatology/40.11.1238 11709607

[11] Salvarani C, Gabriel SE, O’Fallon WM, Hunder GG (1995) Epidemiology of polymyalgia rheumatica in olmsted county, minnesota, 1970–1991. Arthritis Rheum 38(3):369–373. 10.1002/art.1780380311 7880191 10.1002/art.1780380311

